# Phenotypical Characteristics of the Black Yeast *Exophiala dermatitidis* Are Affected by *Pseudomonas aeruginosa* in an Artificial Sputum Medium Mimicking Cystic Fibrosis–Like Conditions

**DOI:** 10.3389/fmicb.2020.00471

**Published:** 2020-03-20

**Authors:** Lisa Kirchhoff, Ann-Kathrin Weisner, Mona Schrepffer, Andrea Hain, Ulrike Scharmann, Jan Buer, Peter-Michael Rath, Joerg Steinmann

**Affiliations:** ^1^Institute of Medical Microbiology, University Hospital Essen, University of Duisburg-Essen, Essen, Germany; ^2^Institute of Clinical Hygiene, Medical Microbiology and Infectiology, Klinikum Nürnberg, Paracelsus Medical University, Nuremberg, Germany

**Keywords:** polymicrobial infections, *Pseudomonas aeruginosa*, *Exophiala dermatitidis*, cystic fibrosis, biofilms, dimorphism, virulence, *Galleria mellonella*

## Abstract

Research into the cooperative pathogenicity of microbes in cystic fibrosis (CF) lungs is crucial for an understanding of the pathophysiology of infections and the development of novel treatment strategies. This study investigated the impact of the common CF-associated bacterial pathogen *Pseudomonas aeruginosa* on the black yeast *Exophiala dermatitidis*. It evaluated the planktonic growth, biofilm formation, morphology, and virulence of the fungus in the presence or absence of *P. aeruginosa*. It also determined the role of *P. aeruginosa* quorum-sensing (QS) molecules within these interactions, e.g., by using sterile culture filtrate and QS-deficient mutants. *P. aeruginosa* is known to inhibit the planktonic growth of *E. dermatitidis*. We found that fungal biofilm formation increased in the presence of *P. aeruginosa* after 24 h but is decreased significantly after 48 h. This effect was reversed when, instead of QS wild-type strains, ΔlasR, and ΔrhlR mutants were added to *E. dermatitidis* biofilm formation. The number and length of hyphae were substantially reduced when *E. dermatitidis* was co-cultivated with *P. aeruginosa*, but not when it was co-cultivated with the mutants. Experiments testing the virulence of *E. dermatitidis* in the greater wax moth *Galleria mellonella* showed a synergetic effect on larval killing when *E. dermatitidis* was injected together with *P. aeruginosa* culture filtrate. Survival rates were decreased when biofilm culture filtrate was added but not when planktonic culture filtrate was added. In summary, *P. aeruginosa* affects the growth, morphology, biofilm formation, and virulence of *E. dermatitidis.* N-acyl-L-homoserine lactone (AHL) QS molecules regulated factors that have been shown to contribute to the inhibition of the ability of *E. dermatitidis* to form filaments and biofilm.

## Introduction

The respiratory tract of cystic fibrosis (CF) patients is commonly colonized by a wide spectrum of microbiota of both bacterial and fungal species. Although some of those organisms are harmful pathogens, such as *Pseudomonas aeruginosa*, *Staphylococcus aureus*, or *Burkholderia cepacia* complex, which are associated with a decline in lung function (Hudson et al., 1993; Courtney et al., 2007), the role of others, including many fungal species, has not yet been clearly distinguished (Hauser et al., 2011). Several studies have examined the virulence of important CF-relevant bacteria, e.g., *P. aeruginosa*. However, less is known about the interactions between bacterial and fungal species within CF lungs, especially their interkingdom communication.

Among the most commonly isolated bacterial pathogens is *P. aeruginosa* (*Pa*), which is believed to be the main cause of respiratory infections (Elkin and Geddes, 2003). *Pa* often dominates the CF lung microbiome in both children and adults; it is present in the lungs of approximately 52% of all CF patients (Hauser et al., 2011). Chronic *Pa* infections among CF patients are commonly caused by biofilm-growing mucoid strains (Bjarnsholt et al., 2009) and are associated with poorer clinical outcome and higher mortality (Blanchard and Waters, 2019). In the past, *P. aeruginosa* has been reported to gain in resistance mechanisms towards antimicrobial therapy, among them the formation of biofilms (Breidenstein et al., 2011).

The black yeast-like fungus *Exophiala dermatitidis* (*Ed*) frequently colonizes the respiratory tract of CF patients; isolation rates range from 1% to 19% (Haase et al., 1990; Bakare et al., 2003; Kondori et al., 2014; Kirchhoff et al., 2019). *Ed* can cause systemic infections (Kondori et al., 2014). It belongs to the melanized fungi and is characterized by its dimorphic character (De Hoog and Guarro, 1995). Its virulence potential has been recently demonstrated in an *in vivo* model using the greater wax moth *Galleria mellonella* (Olsowski et al., 2018). *Ed* can also form biofilms, which contribute to its resistance to anti-infective therapy (Kirchhoff et al., 2017).

Biofilm formation is one important factor contributing to a pathogen’s virulence potential and is important for human health, especially among CF patients (Donlan and Costerton, 2002). Thus, biofilm formation studies have been of increasing interest in recent years, as have studies dealing with microbes in their sessile forms (Kolter and Greenberg, 2006). Several studies on anti-biofilm agents are published, especially focusing on bacterial biofilm, e.g., the anti-biofilm peptide 1018, effective against *P. aeruginosa* biofilms (de la Fuente-Núñez et al., 2014). Biofilms are defined as differentiated, homogeneous masses of microbes that form on surfaces and are surrounded by an extracellular matrix (ECM) with open water channels. The gene expression of biofilm-associated cells is different from that of planktonic cells (Donlan and Costerton, 2002). Important for biofilm regulation processes is the expression of quorum-sensing (QS) genes. QS is important for the communication between microorganisms via chemical signal molecules. These autoinducers are produced and released by pathogens in relation to cell population densities (Waters and Bassler, 2005). One well-studied QS system is the N-acyl-L-homoserine lactone (AHL) QS system in *Pa*. The AHL QS system is associated with two well-studied QS pathways: the rhl pathway and the las pathway, both of which (auto-)induce several virulence genes, e.g., LasB, LasA, RhlAB, Pyocyanin, Pyoverdine, and ToxA. The autoinduction molecule of the las pathway is N-3-oxo-dodecanoyl-L-homoserine lactone (3-oxo-C12 HSL); that of the rhl pathway is N-butanoyl-L-homoserine lactone (C4-HSL) (Smith and Iglewski, 2003). It is known that these QS molecules influence the phenotypical characteristics of *Candida albicans* (Hogan and Kolter, 2002). The study reported here investigated the interactions between the CF-relevant pathogens *Pa* and *Ed* and identified the role of *Pa* AHL QS molecules in these interactions.

## Materials and Methods

### Strains

The bacterial and fungal strains used in this study are listed in Table 1. We analyzed three *Ed* isolates: one isolate from the sputum of a CF patient and two invasive strains isolated from Asian patients. All were reference strains: Centraalbureau voor Schimmelcultures (CBS) 109154, CBS 116372, and CBS 552.90. *Pa* strain Pa7 (DSM 1128) and Pa14 ΔlasR and ΔrhlR, as well as their corresponding wild type (WT; DSM 19882), were included.

**TABLE 1:** Bacterial and fungal strains used in this study.

Strain	Relevant features	Reference or Origin
**Ed**		
P1	Invasive isolate	CBS 109154
P2	Invasive isolate	CBS116372
CF2	CF sputum isolate	CBS 552.90
**Pa**		
Pa7	WT strain	DSM 1128
Pa14 WT	WT strain	DSM 19882
Pa14 ΔlasR	LasR knockout mutant of Pa14 WT	Lorenz et al., 2019
Pa14 ΔrhlR	RhlR knockout mutant of Pa14 WT	Brouwer et al., 2014

*CBS, Centraalbureau voor Schimmelcultures; CF, cystic fibrosis; DSM, Deutsche Stammsammlung; Ed, Exophiala dermatitidis; Pa, Pseudomonas aeruginosa; WT, wild-type.*

### Cultivation

Both bacterial and fungal isolates were cultivated in liquid culture before being used in further assays. We grew *Ed* for 48 h in Sabouraud broth (Sab) containing 2% glucose at 35°C under rapid shaking (200 rpm). *Pa* was cultivated overnight in lysogeny broth (LB) at 36°C under moderate shaking (140 rpm). Cells were washed three times with sterile phosphate-buffered saline (PBS) before further use.

### Planktonic Growth Assay

Planktonic growth of *Ed* and *Pa* was estimated in mono- and co-culture in a CF sputum condition. An artificial sputum medium (ASM; pH 6.9), modified after Kirchner et al. (2012), was used (Table 2).

**TABLE 2:** Artificial Sputum Medium (ASM).

Ingredient	Amount (g/L)	Supplier
Fish sperm DNA (low molecular weight)	4.0	Sigma, Steinheim, Germany
Mucin from porcine stomach (type II)	5.0	Sigma, Steinheim, Germany
DTPa	0.0059	Sigma, Steinheim, Germany
NaCl	5.0	NA
KCl	2.2	NA
Casamino acids	5.0	BD, New Jersey, United States
Egg yolk emulsion	5.0 mL	Sigma, Steinheim, Germany
1 M Tris (for pH adjustment)	as necessary	NA

*The recipe was modified from that used by Kirchner et al. (2012). The fish sperm DNA and the mucin were dissolved overnight, each in 250 mL sterile water at 4°C. The casamino acids were dissolved in 150 mL sterile water, and the diethylenetriaminepentaacetic acid (DTPa), NaCl, and KCl were dissolved together in 100 mL water. Solutions 1 through 4 were pulled together, and the pH was set to 6.9 by the addition of 1 M Tris. Water was added up to a volume of 1 L, and the solution was sterile filtered. After sterilization, the egg yolk emulsion was added. The prepared medium was stored at 4°C for as long as 1 month.*

The medium was filled to a volume of 25 mL in a 100-mL Erlenmeyer flask. Inoculum was set to 1 × 10^6^ cells per mL for *Ed* and 1 × 10^7^ cells per mL for *Pa*, equaling a fungus to bacteria ratio of 1:10. Incubation was carried out at 36°C and 140 rpm, ensuring aeration. For growth detection, cell count was estimated for t_0_, t_2_, t_4_, t_6_, t_8_, t_24_, and, for cultures including fungi, additionally at t_48_ and t_72_, for which t_0_ was set at 0.5 h after inoculation so that the cells could become accustomed to the conditions. For cell count determination, various dilutions of the culture in sterile PBS were plated on selective agar plates suitable for the organisms used. Next, 100 μL of the suspension was plated onto each agar plate, and the plates were incubated for 24 to 72 h until countable colonies appeared. Colony-forming units (CFU) per mL were counted.

Additionally, a disk-diffusion assay on RPMI medium (Oxoid, Wesel, Germany) was carried out according to European Committee on Antimicrobial Susceptibility Testing (EUCAST) recommendations (Antimicrobial susceptibility testing EUCAST disk diffusion method, Version 7, 2019) with some modifications. A cell suspension of *Ed* in NaCl with a McFarland standard of 2 was prepared and spread evenly onto an RPMI agar plate by swabbing in three directions. Disks (Becton, Dickinson, and Company, Franklin Lakes, NJ, United States) were impregnated with the dilutions. The disks were allowed to remain at room temperature until the diluent had completely evaporated. Disks loaded with culture filtrates or live *Pa* cells, in concentrations of 10^6^ cells/mL, were firmly placed onto the surface of the agar within 15 min after inoculation. Plates were incubated for 72 h at 35°C until a cell layer appeared. Disks impregnated with voriconazole (64 μg/ml) and paper disks impregnated with PBS were used as controls. Tests were performed in duplicate.

### Biofilm Formation Assay

The biofilm-forming ability of *Ed and Pa* isolates was analyzed with a crystal violet (CV) assay as described elsewhere (Kirchhoff et al., 2017) and by CFU counts after biofilm detachment for species-specific quantification purposes. Briefly, a suspension in ASM (Table 2) was set with a cell density of 1 × 10^6^ cells per mL for the fungi and 1 × 10^7^ cells per mL for the bacteria, equaling a fungi-bacteria ratio of 1:10. Aliquots (200 μL each) of the suspension were added to each well of a sterile polystyrene flat-bottomed 96-well microtiter plate. Sterile ASM was used as a control for a blank correction. Suspensions with the single species separately (monoculture) and with both species together (co-culture) were prepared and used in the biofilm formation assay.

After incubation over a period of 24 to 48 h at 36°C without agitation, the plate was rinsed thrice with PBS. For the CV stain assay, 125 μL of a 0.1% CV solution was added to each well. The staining was carried out for a minimum of 20 min at room temperature. Three additional rinsing steps with PBS were followed by air drying overnight. Next, 200 μL of 30% acidic acid was added to each well of the plate and incubated for 30 min, after which 150 μL of the solution was transferred from each well to a fresh microtiter plate. The plate was read at an optical density of 620 nm (OD_620_). The OD_620_ of each well was calculated by subtraction of the blank reading. An inoculum size was estimated by CFU counting as follows: 100 μL of suspension at various dilutions was plated on malt extract agar, and the grown colonies were counted after incubation for 48 h at 35°C.

In addition, Transwell permeable supports were used to examine the role of direct cell-cell contact for interaction processes. Transwell permeable supports are devices suitable for *in vitro* studies of transport and metabolic activity. In this assay, polyester (PET) membranes 6.5 mm in diameter with a pore size of 0.4 μm were used as inserts (Costar 3470 clear, Corning Incorporated, Corning, NY, United States), preventing the cells in the insert from penetrating into the well. The target species was added to the well, whereas the species of interest was added to the insert. Biomass in the biofilm within the well was measured by CV staining, as described above.

In addition to CV-staining methods, biofilm detachment and subsequent CFU counts were carried out for quantification of the species-specific cell counts within the polymicrobial biofilm communities. For biofilm detachment, 0.1% dithiothreitol (Sputasol, Oxoid, Wesel, Germany) was added to the biofilm and incubated for 15 min at room temperature under slight agitation. Pipetting up and down rendered the biofilm easily detachable, and the number of CFU on selective agar was estimated.

To detect the effect of the lasR QS system, we prepared lasR-related signal molecule 3-oxo-C12 HSL (Sigma, Steinheim, Germany) in a stock solution of 10 mM in DMSO. A concentration gradient from 0.19 to 100 μM was added to a 96-well microtiter plate. As a control, medium without agent was used. DMSO in corresponding concentrations between 0.0019 and 1% has been added to biofilm formation and used as the control.

Next, the co-culture biofilm assays were repeated under anaerobic conditions. Biofilm was formed as described above. Microtiter plates were stored in an anaerobic GasPak EZ Gas Generating Container System (Becton, Dickinson, and Company). Three GasPak packages were used for each container. After the container was closed, hypoxia was achieved after approximately 20 min. After each opening of the container, new GasPaks were necessary. Analysis of biofilm formation was carried out as described above.

### Preparation of Culture Filtrates

Sessile and planktonic *Pa* cultures were cultivated as described above. Sterile filtrates were produced by centrifugation at 3,500 × g for 10 min, repeated twice. The supernatant was sterile-filtered using a filter with a pore size of 0.22 μm. Sterile culture filtrates were applied at various volumes according to the corresponding cell count within the culture. The pH of culture filtrates was controlled before use, ensuring a pH between 6.8 and 7, which equals the pH of ASM.

### Ed Morphology Assay

The morphology of *Ed* in the presence of *Pa* wild-type, QS mutants and their culture filtrates was estimated. Additionally, 3-oxo-C12 HSL (100 μM), prepared as described above) and a DMSO (1%) control was included in the morphology assay. During cultivation in mono- or co-culture, morphology was observed at various time points and was visualized via fluorescence microscopy. In addition, the length of hyphae was measured. For both purposes, cells were washed in PBS and stained with calcofluor white (Becton, Dickinson, and Company) for further fluorescence microscopy. Image acquisition and analysis were carried out with a Zeiss Axio LabA1 microscopy system and Zen 2 core imaging software (V2.5; Zeiss, Jena, Germany).

### Biofilm Microscopy and Biofilm Thickness

Biofilms were visualized with confocal laser scanning microscopy (CLSM). Biofilms were formed as described above over a period of 48 h in glass chambers with 1 μ wells (ibidi, Martinsried, Germany). After 48 h of incubation at 36°C, the biofilms were washed with PBS and dried. Next, the biofilms were fixed by incubation with 100% methanol for 2 min, and the DNA was stained for 3 min with acridine orange (Becton, Dickinson and Company). The stained biofilm was washed twice with PBS before microscopy. A laser with a wavelength of 488 nm was used. We generated both 2.5-D images and 2-D images. Mono- and co-culture biofilms of *Ed* were analyzed.

CLSM was also used for measurements of the thickness of the ECM of the biofilms. ECM thickness was determined by staining with Invitrogen FilmTracer SYPRO ruby biofilm matrix stain (Thermo Fisher Scientific, Waltham, MA, United States) and subsequent CLSM. Biofilms were formed as described above over a period of 24, 48, or 72 h in 1 μ-well glass chambers (ibidi). After incubation at 36°C, the biofilms were washed once with PBS and dried. Then the biofilms were fixed by incubation with 100% methanol for 2 min, and the proteins in the ECM were stained for 30 min with Invitrogen FilmTracer SYPRO ruby biofilm matrix stain (Thermo Fisher Scientific). The stained biofilm was washed once with sterile water, and 0.5 μL was left in the well before microscopy.

For image acquisition, a laser with a wavelength of 405 nm was used. CLSM was performed with a 40 × objective (LSM 710, Zeiss, Jena, Germany). For the analysis of the various mono- and co-cultures, three independent biofilm experiments were performed. In all three rounds, each sample well was analyzed with five image stacks acquired at various time points (24, 48, or 72 h). Images were acquired from random positions in the well at a distance of at least 2 μm from the edges. The first and last images of the Z-stack were acquired at the first and last visualized ECM. The thickness of the ECM was calculated by the number of images and the intervals (in μm) between them. Images were analyzed with Zen black software (Zeiss, Jena, Germany) and Image J software (National Institutes of Health, Bethesda, MD, United States).

### Effect of Pa QS Molecules on Ed Biofilm Formation

Biofilm was formed in polymicrobial cultures containing *Ed* and the *Pa* WT, ΔlasR, or ΔrhlR. In addition, biofilm was formed in medium containing 3-oxo-C12 HSL at various concentrations (0.195–100 mM). Three *Ed* isolates were tested.

### *In vivo* Virulence

Virulence in mono- and co-cultures of *Ed* and *Pa* was investigated with the alternative infection model *G. mellonella.* Survival analysis in the insect model was carried out as previously described for *Ed* (Olsowski et al., 2018). For *Ed*, an inoculum of 1 × 10^8^ cells per mL was prepared in PBS. In contrast, for *Pa*, an inoculum of 1 to 10 cells per larva was lethal. Thus, for coinfection with *Ed* and *Pa*, we used *Pa* sterile culture filtrates and biofilm filtrates. Filtrate was added in volumes corresponding to 1 × 10^9^ cells per mL, equaling a theoretical ratio of fungus to bacteria of 1:10. Before *G. mellonella* caterpillars were infected, they were maintained for 48 h at 22°C in the dark, thereby ensuring their health and fitness. Health was assessed by body color: yellow-cream–colored larvae were included in the experiments. Larvae were weighed and classified by weight into 3 groups: 200 (± 50) mg; 300 (± 50) mg; and 400 (± 50) mg. The area of injection was disinfected with a sterile cotton swab saturated with Cutasept F (Bode Chemie GmbH, Hamburg, Germany). Caterpillars were infected by the injection of cell solution directly into the hemocoel with piercing of one of the last prolegs. A volume of 10 μL per 300 mg larvae was injected with a syringe pump (SyringePumpPro model LA-100; Landgraph Laborsysteme HLL GmbH, Langerhagen, Germany) at an injection rate of 2 μL/sec. Non-infection control caterpillars were injected with sterile PBS. Additionally, a non-injection control was included. Each sample included 10 larvae.

Infected caterpillars were incubated at 37°C and were observed daily for up to 14 d for monitoring of viability and fitness of larvae. Dead caterpillars were removed from incubation, and *Ed* was recultivated from the caterpillars’ tissue by mechanical lysis of dead larvae: The dead larvae were disinfected in 70% ethanol for at least 5 min before they were placed into FastPrep lysis M tubes (MP Biomedicals, LLC, Santa Ana, CA, United States) containing 500 μL PBS. The prepared tubes were inserted into the MagNa Lyser (Roche, Grenzach-Wyhlen, Germany) for 15 sec at 7,000 × g. The lysis product was cultivated on suitable selective agar and examined by eye after incubation for 24 to 72 h. For identification of distinct species, we used matrix-assisted laser desorption/ionization time of flight (MALDI TOF) mass spectrometry (bioMérieux, Nürtingen, Germany). Each experiment was repeated three times.

### Statistical Analysis

Unless otherwise stated, values are presented as the mean obtained from three separate observations. Values were compared with Student’s *t*-test; statistical significance was set at the level of *P* < 0.05. Statistical analysis was performed with GraphPad Prism 6 (GraphPad Software Inc., La Jolla, CA, United States).

## Results

### *P. aeruginosa* Inhibits *E. dermatitidis* Planktonic Growth

**FIGURE 1:**
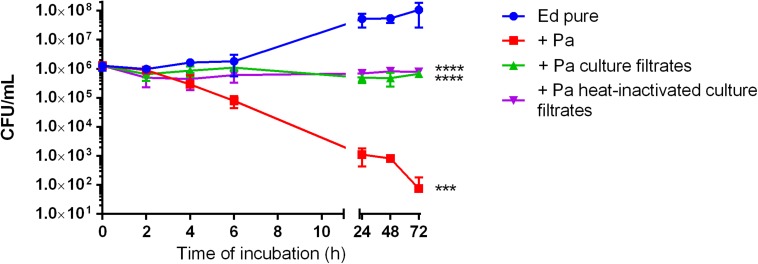
Growth of *Exophiala dermatitidis* in pure artificial sputum medium (ASM) (Ed pure) and in co-cultivation with *Pseudomonas aeruginosa* (Pa, PA07), as well as in ASM with sterile, heat-inactivated culture filtrates. Growth was detected by counting colony-forming units (CFU) during cultivation at 35°C under slight agitation. Two-way ANOVA, ***P < 0.001, ****p < 0.0001, *N* = 3.

To evaluate the effect of *Pa* on *Ed* planktonic growth, we performed growth assays of three *Ed* strains (P1, P2, and CF2) in mono- and co-culture with *Pa* WT and QS-deficient mutant strains (ΔlasR and ΔrhlR). Growth was determined by counting CFU in liquid culture at 35°C with slight agitation over a period of 24 h. Co-cultivation of *Ed* with *Pa* WT inhibited *Ed* planktonic growth. After 24 h of cultivation, the number of detectable *Ed* cells was significantly reduced (two-way ANOVA, *p* = 0.0028), whereas pure *Ed* cultures contained a concentration of 1 × 10^8^ cells/mL. An inhibitory effect was also seen when *Ed* was cultivated together with heat-inactivated *Pa* cells or sterile culture filtrates (Figure 1).

**FIGURE 2:**
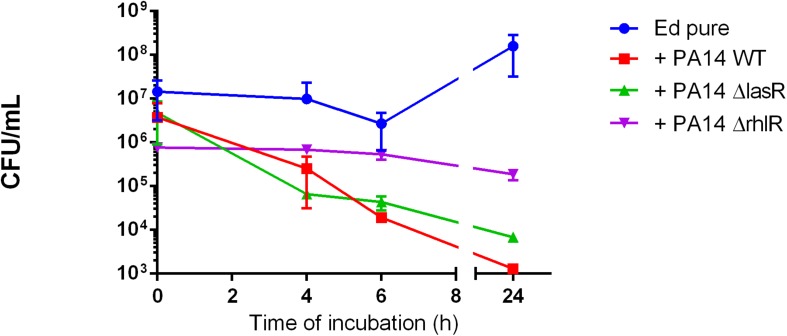
*Exophiala dermatitidis* (Ed) growth in mono- (Ed pure) and co-cultures with a wild-type strain of *Pseudomonas aeruginosa* (Pa; PA14 WT) and with QS-deficient mutants (PA14 ΔlasR and PA14 ΔrhlR). *N* = 3.

Furthermore, we found no significant difference in number of detectable *Ed* cells between *Pa* WT and the QS-deficient mutant strains ΔlasR and ΔrhlR; each PA strain addition resulting in growth inhibition of *E. dermatitidis* (Figure 2).

In an additional disk-diffusion assay, we confirmed the growth inhibitory effect of *Pa* on *Ed*. The disk-diffusion assay is commonly used as a routine laboratory diagnostic procedure for testing antimicrobial susceptibility. Target substances were applied to disks and placed on an agar plate with the seeded microbial species of interest; after growth, the inhibitory zone was measured. The inhibition zone for *Pa* WT strain PA07 was 1.2 cm, whereas that for the control was 0 cm. In contrast, PA planktonic- and biofilm culture filtrate did not inhibit *Ed* growth in disk-diffusion assays (Supplementary Figure S1).

### *P. aeruginosa* Inhibits *E. dermatitidis* Biofilm Formation via Secretion of QS Molecules in a Time-Dependent Manner

**FIGURE 3:**
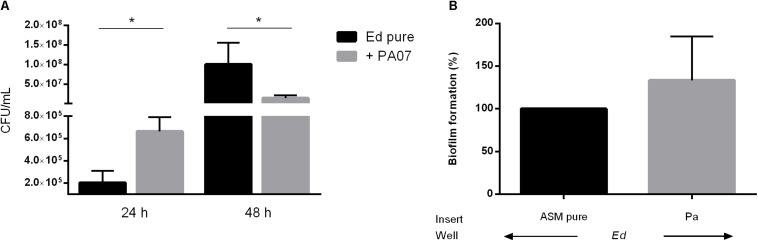
*Exophiala dermatitidis* (Ed) biofilm formed after 24 or 48 h at 36°C in artificial sputum medium (ASM). **(A)** Biofilms formed in mono- (Ed pure) and co-cultures with *Pseudomonas aeruginosa* (Pa). Biofilms were estimated by detachment of biofilm with 0.1% dithiothreitol and subsequent count of colony-forming units (CFU per mL). **(B)** Biofilms in Transwell permeable support. Ed suspension for biofilm formation was placed into the well, whereas Pa was placed in the insert. As a control, pure ASM was used. Unpaired *t*-test, *P < 0.05, *N* = 3.

In addition to the planktonic form of life, we also investigated the sessile form of *Ed* in the presence or absence of *Pa*. Additionally, we investigated the effect of *Pa* culture filtrates from planktonic and biofilm cultures on *Ed* biofilm formation. Fungal biofilm formation in a duo-culture biofilm was quantified after detachment of biofilm structures by incubation with 0.1% dithiothreitol (DTT); subsequently, CFU were counted. We also investigated *Ed* biofilm formation when direct cell-to-cell contact between *Ed* and *Pa* was inhibited by the use of transwell-permeable supports. Biofilm formation by *Ed* was significantly (paired *t*-test; *p* = 0.0285) increased when *Ed* was grown together with *Pa* WT for 24 h (Figure 3A). A slight increasing effect was also detected when direct cell-to-cell contact was excluded (Figure 3B). However, this difference was not statistically significant (paired *t*-test, *p* = 0.3761).

In contrast, after 48 h we detected a significant (paired *t*-test, *p* = 0.0167) decrease in *Ed* biofilm formation in the presence of *Pa* as demonstrated for PA07 (Figure 3A). This effect was strain-dependent as PA14 did not show significant inhibitory effects on *Ed* biofilm after 48 h.

**FIGURE 4:**
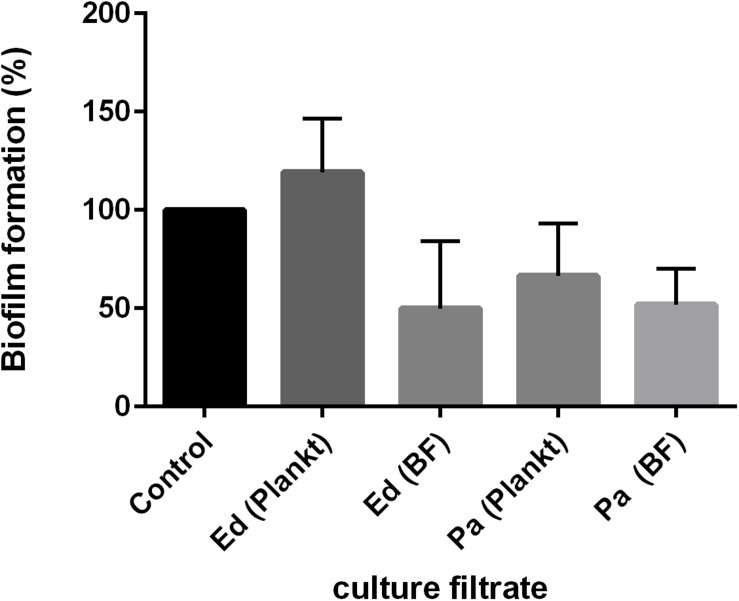
*Exophiala dermatitidis* (Ed) biofilm formation after 48 h at 36°C with culture filtrates of planktonic (plankt) or biofilm (BF) culture from *Pseudomonas aeruginosa* (Pa). Also, Ed culture filtrate was added. As a control, biofilm formation in pure ASM was assessed. *N* = 3.

Both effects were also detected when the culture filtrate of *Pa* was used and under the exclusion of cell-to-cell contact. Remarkably, after 48 h of biofilm formation, *Pa* planktonic culture filtrate was less inhibitory on *Ed* biofilm formation than was *Pa* biofilm culture filtrate (Figure 4). The effect of *Pa* biofilm culture filtrate on *Ed* biofilm formation (a reduction of 40–50%) was similar to that of *Ed* biofilm culture filtrate.

**FIGURE 5:**
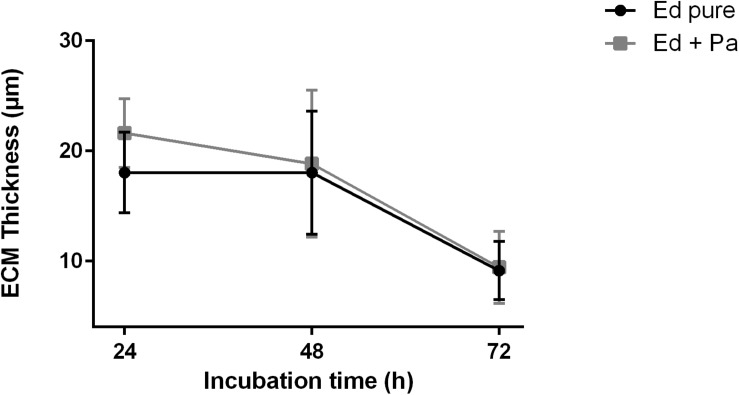
Extracellular matrix (ECM) thickness (μm) of *Exophiala dermatitidis* mono- (Ed pure) and co-culture biofilms with *Pseudomonas aeruginosa* (Pa). *N* = 10.

Additional observations of biofilm ECM thickness over time confirmed the results of biofilm assays. CLSM was carried out after 24, 48, and 72 h of biofilm formation, and Z-stack images were obtained (Supplementary Figure S2). After 24 h, ECM was thicker in *Ed* co-culture than in *Ed* monoculture (Figure 5). After 48 and 72 h we found a decrease in ECM thickness for both mono and co-culture, and we could detect no difference in ECM thickness between the cultivation set-ups. However, the differences were not statistically significant.

**FIGURE 6:**
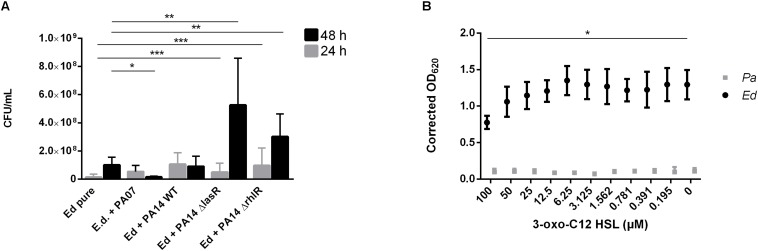
*Exophiala dermatitidis* (Ed) biofilms formed after 24 h (gray) or 48 h (black) at 36°C in artificial sputum medium (ASM). **(A)** Biofilms in mono- (Ed pure) and co-culture with *Pseudomonas aeruginosa* (Pa) wild-type (WT) strains PA07 and PA14, as well as two quorum-sensing (QS) mutants lacking LasR and RhlR. Biofilms were estimated by detachment of biofilm with 0.1% dithiothreitol and subsequent count of colony-forming units (CFU per mL). **(B)** Biofilms of *Exophiala dermatitidis* (Ed, P2) and *P. aeruginosa* (Pa, PA14 WT) after 24 h of incubation at 35°C in ASM treated with N-(3-oxododecanoyl)-L-homoserine lactone (3-oxo-C12-HSL) in a concentration gradient from 0 to 100 μM in DMSO. Biomass in biofilm was measured by staining with 0.1% crystal violet. For correction of biomass in biofilm, difference between the DMSO and the DMSO plus 3-oxo C12 HSL treated biofilms has been estimated and subtracted from the positive control. OD, optical density. Unpaired *t*-test, *P > 0.05; **P < 0.01; ***P < 0.001. *N* = 3.

To detect a possible role of the AHL QS molecules in the impact of *Pa* on the formation of *Ed* biofilms, we used two QS-deficient mutants, one with a disruption in the lasR QS system and one with a disruption in the rhlR QS system. In contrast to our findings with WT *Pa*, we detected no inhibitory effect after 48 h when biofilms were formed in the presence of *Pa* Δ*lasR* or Δ*rhlR.* Both QS-deficient mutants caused a significant (unpaired *t*-test; ΔlasR *p* = 0.0057 ΔrhlR *p* = 0.0079) increase in *Ed* biofilm formation (Figure 6A). In contrast, *Ed* did not affect *Pa* biofilm formation (Supplementary Figure S3).

We demonstrated the role of *Pa* QS systems in the described interactions by using *Ed* biofilm assays in which a concentration gradient of 3-oxo-C12 HSL was added. The addition of the lasR synthesis product in increasing concentrations resulted in decreased formation of *Ed* and *Pa* biofilms (Figure 6B). A significant fungal biofilm reduction has been detected for incubation with 100 μM 3-oxo-C12 HSL.

**FIGURE 7:**
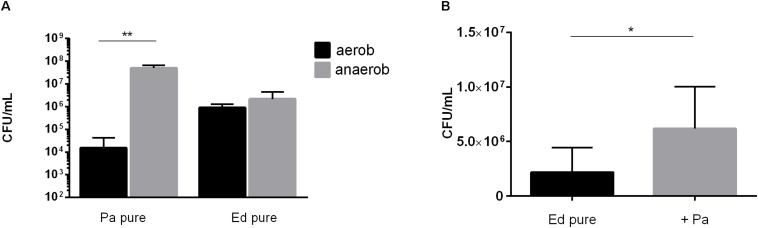
**(A)** *Pseudomonas aeruginosa* (Pa) and *Exophiala dermatitidis* (Ed) biofilm formation in monocultures (pure) after 48 h of biofilm formation at 35°C under aerobic (black) or anaerobic (gray) conditions. Biofilms were quantified after biofilm detachment with 0.1% dithiothreitol. **(B)** Ed biofilm formed after 48 h at 36°C in ASM under hypoxic conditions in monoculture (ED pure) or co-culture with Pa. Biofilms were estimated by detachment of biofilm with 0.1% dithiothreitol and subsequent count of colony-forming units (CFU per mL). Paired *t*-test, *P < 0.05; **P < 0.01, *N* = 3.

Next, we assessed the ability of *Ed* to form biofilm in the presence of *Pa* WT strains under anaerobic conditions and found that biofilm formation was higher under these conditions than under aerobic conditions. Furthermore, we detected a significant (paired *t*-test, *p* = 0.0483) increase in co-cultured fungal biofilm after 48 h (Figure 7).

### *E. dermatitidis* Morphology Is Influenced by the *P. aeruginosa* QS System

**FIGURE 8:**
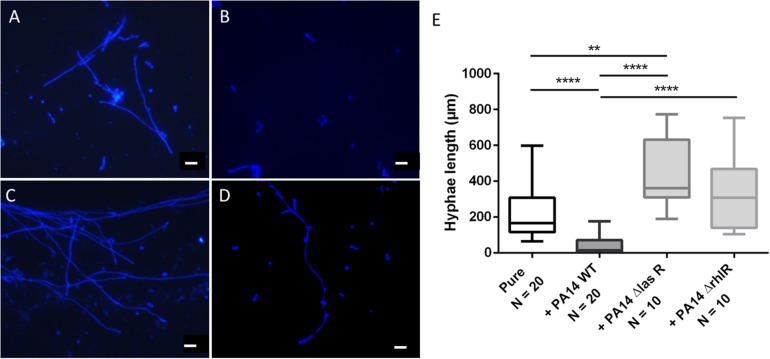
*Exophiala dermatitidis* P2 morphology after 24 h of incubation in monoulture (**A**, pure) or in co-culture with wild-type (WT) *Pseudomonas aeruginosa* (**B**, PA14) or with QS-deficient mutants (**C**, PA14 ΔlasR; **D**, PA14 ΔrhlR). Cultures were stained with calcofluor white and observed with a fluorescence microscope at 365 nm. Rulers indicate a length of 20 μm. **(E)** length (μm) of hyphae of *E. dermatitidis* in mono- (pure) or co-cultures with *P. aeruginosa*. Unpaired *t*-test, **P < 0.01; ****P < 0.0001.

The morphology of dimorphic fungi, especially their switch from the yeast to the hyphal state, is known to be a virulence factor and to be crucial for biofilm formation. Thus, we assessed *Ed* morphology in mono- and co-culture with *Pa.* The main aims of the morphology assays were to estimate the number and length of formed hyphae, thereby detecting morphologic changes caused by co-cultivation with *Pa*. Furthermore, we investigated the role of the *Pa* QS system. The presence of *Pa* cells or *Pa* filtrate resulted in alterations in the morphology of *Ed.* After 24 h of incubation in pure culture, *Ed* was mainly present in the form of pseudo-hyphae and true hyphae. In contrast, when grown in co-culture with *Pa*, most *Ed* cells were present as conidia, either clumped together or as single cells. These cell agglomerates were also present when *Ed* was grown in *Pa* filtrate (Supplementary Figure S4). The formation of hyphae was impaired when *Ed* was grown together with *Pa*, even under filament-inducing conditions (0 rpm). This effect was reversed when an AHL QS knock-out *Pa* strain, either ΔlasR or ΔrhlrR, was used. When *Ed* was grown together with PA14 ΔlasR, the number of filaments was larger than when *Ed* was grown in pure culture. The inhibition of filament formation also affected the length of hyphae, rendering them substantially shorter (Figure 8). These effects could have been demonstrated as well by *Pa* culture filtrates (Supplementary Figure S5).

We measured the length of hyphae and found that they were significantly (unpaired *t*-test, *P* < 0.0001) shorter when *Ed* was grown in co-culture with WT *Pa*. Remarkably, hyphae were longer when *Ed* was co-cultivated with *Pa* strains deficient in LasR and RhlR than when they were cultivated together with the WT strain. Additionally, hyphae were significantly (unpaired *t*-test, *p* = 0.0008) longer when *Ed* was co-cultivated with a lasR knockout *Pa* strain than when *Ed* was cultivated in pure culture. Culture filtrates of *Pa* WT exhibited filament inhibitory effects on *Ed* comparable to those described for viable *Pa* cultures, resulting in significantly reduced hyphae length (Supplementary Figure S4). 3-oxo C12 HSL added in concentrations of 100 μM showed comparable filament inhibiting effects on *Ed.* A DMSO control did not show any effect on *Ed* morphology. In contrast to *Pa* WT, culture filtrates of QS mutants ΔlasR and ΔrhlR did not affect *Ed* morphology. However, a filament enlarging effect could not be detected either.

The pH of the media used also affected the morphology of *Ed*. For filament formation, we found an association between decreases in pH and increases in the length of hyphae (Supplementary Figure S6). Control of pH of 24 h old cultures revealed increased pH from 7 to 8 when *Ed* was cultured in presence of *Pa* cells (Supplementary Table S1). No differentiation could be made between *Pa* WT and QS deficient mutants. In contrast, *Pa* culture filtrates did not increase *Ed* culture pHs, even though similar effects of culture filtrates and viable *Pa* cultures on *Ed* morphology were detected. Thus it is assumed that the altered pH is not entirely responsible for the detected filament formation inhibition.

### Virulence of *E. dermatitidis* Is Increased by *P. aeruginosa in vivo*

In addition to the *in vitro* analysis of the impact of *Pa* on *Ed*, the present study also included *in vivo* virulence assays using the alternative non-invertebrate infection model *G. mellonella* to estimate the effect of *Pa* on the virulence of *Ed*. The survival of *G. mellonella* was monitored for 14 days after infection. As a non-infection control, PBS was injected. The infection of *G. mellonella* with *Pa* resulted in lethal doses of fewer than 10 cells per larva; the values are lower for PA14 than for PA07 (Supplementary Figure S7). Thus, we investigated the virulence potential of co-infection with *Ed* and *Pa* by applying sterile culture filtrates instead of viable cells. Survival analysis showed that *Pa* culture filtrate exerted a substantially effect on the virulence of *Ed* in *G. mellonella*. Both biofilm and planktonic culture filtrate of *Pa* increased the killing of *Ed*-infected larvae. A reduction in survival of 50% was achieved after 3 to 6 days for planktonic PA07 culture filtrate and after 1 to 3 days for PA07 biofilm filtrate, whereas *Ed* alone killed 50% of larvae after 4 to 5 days. The virulence-increasing effect on *Ed* was greater when sessile culture filtrate was added compared to planktonic culture filtrate.

**FIGURE 9:**
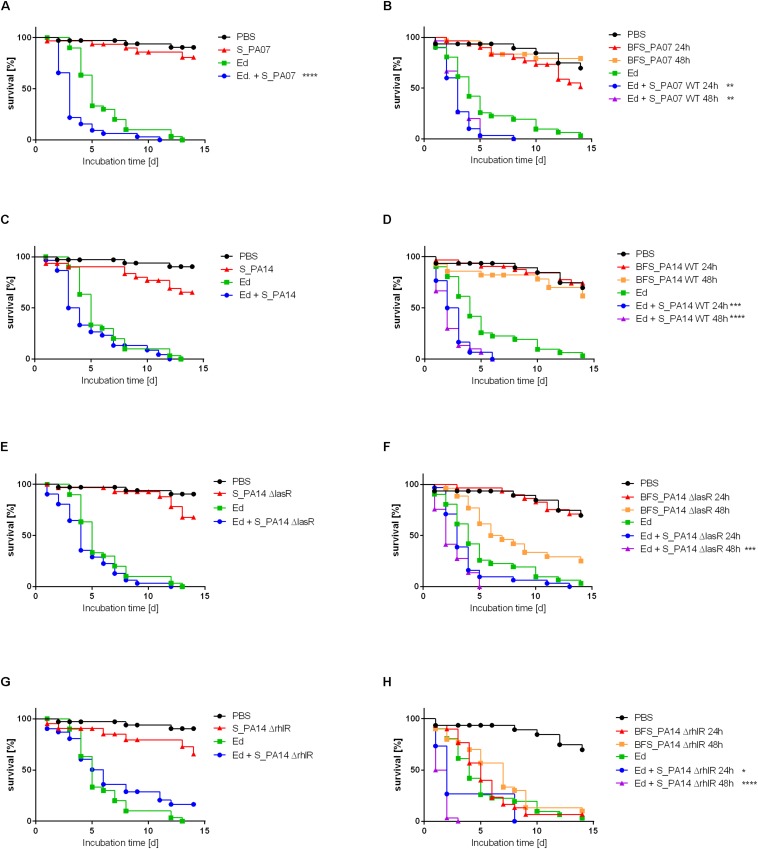
Survival of *Galleria mellonella* after infection with *Exophiala dermatitidis* (Ed; Strain P2) with or without *Pseudomonas aeruginosa* (Pa) culture filtrate of strain PA07 (A/B), PA14 wild-type (wt; C/D), PA14 ΔlasR (E/F), and PA14 ΔrhlR (G/H). As a control, sterile phosphate-buffered saline (PBS) was used. **(A, C, E, G)** Planktonic culture filtrate was added to injection suspension (S). **(B, D, F, H)** Biofilm culture filtrate (BFS) 24 or 48 h old was added to the injection suspension. *N* = 30. Log-rank (Mantel-Cox) test for statistical significance: *P < 0.05; **P < 0.01; ***P < 0.001; ****P < 0.0001.

The survival rate of insects was substantially lower when they were infected with *Ed* in the presence of *Pa* filtrate than injected with pure *Ed*. This *Ed* virulence benefiting effect was seen for culture filtrate from both *Pa*WT and QS deficient mutants (Figure 9).

The pure biofilm culture filtrates of the *Pa* mutant lacking RhlR exerted a killing effect on the larvae that was similar to the killing effect of *Ed* pure injection. In contrast, planktonic culture filtrates of PA14ΔrhlR were less virulent: survival rates of *Galleria* infected with these culture filtrates were comparable to those associated with the WT and the ΔlasR mutant strains. This finding was the same for all *Ed* strains included in this study (P2: Figure 9; P1, CF2: Supplementary Figures S8, S9).

## Discussion

Previous studies found relevant interactions between various CF-associated pathogens. However, unlike the more common CF-associated pathogens *Pa*, *C. albicans*, and *Aspergillus fumigatus*, less is known about the cooperative pathogenicity of *Ed* and other CF-relevant microbes. This is the first study on combined effects of the CF-relevant bacterium *Pa* on the black yeast-like fungus *Ed*. The results show that *Pa* QS molecules have an effect on *Ed*.

*Pa* is known to exhibit two well-studied QS systems. One is the *las* system, with the AHL signal molecule 3-oxo-C12 HSL and LasR as the transcriptional regulator protein. The second is the *rhl* system, with RhlR as the transcriptional regulator protein and C4-HSL as the signal molecule (Smith and Iglewski, 2003). Noticeably, 3-oxo-C12 HSL diffusion out of bacterial cells is slower than that of C4-HSL (Smith and Iglewski, 2003). AHL-QS molecules, particularly 3-oxo-C12 HSL, secreted by *Pa* are reported to influence the morphology of *C. albicans*, inhibiting the formation of *C. albicans* filaments, thereby impairing biofilm formation (Hogan et al., 2004; De Sordi and Mühlschlegel, 2009; Holcombe et al., 2010; Bandara et al., 2013; Bergeron et al., 2017). Furthermore, it is known that *Pa* interacts with *A. fumigatus* in inhibiting fungal growth and biofilm formation (Sass et al., 2019). A *Pa* phenotype and a source-dependent effect have been identified: CF isolates are more inhibitory than non-CF isolates, and non-mucoid isolates are more inhibitory than mucoid isolates (Ferreira et al., 2015). The inhibitory effect was also detected for *Pa* culture filtrates, demonstrating that no contact with live cells is necessary for interaction (Ferreira et al., 2015). Ferreira et al. (2015) also found that the inhibitory effect of *Pa* sessile filtrates is higher than that of planktonic culture filtrates, and Mowat et al. (2010) found that the effect of *Pa* QS mutants is smaller than the effect of wild-type strains.

In previous work it has been shown that AHL bacterial QS molecules are directly recognized by eukaryotic cells (Dudler and Eberl, 2006). It is also known that some fungi produce AHL antagonists (Rasmussen et al., 2005). While the existence of fungal QS systems have been proven, none of the bacterial known QS systems could be found in eukaryotes, implicating fungi QS systems evolved differently (De Sordi and Mühlschlegel, 2009). The response of *Ed* to *Pa* QS molecules implicates the confirmation of following requirements: The signal is secreted by *Pa* in a physiological relevant concentration. *Ed* is capable to recognize the signals and to respond specifically and the concentration of signal molecule is not toxic to *Ed* (Monds and O’Toole, 2008). Response on QS recognition is suggested to be among others the inhibition of gene expression encoding for adhesion and filament formation and relevant signal cascades. On molecular level, only little is known on the mechanisms of action of QS in fungi. For *C. albicans*, 3-oxo-C12 HSL has been shown to influence morphogenesis by inhibition of the Ras1-cAMP/protein kinase A (PKA) signaling pathway (Davis-Hanna et al., 2007). Clinical isolates of *Pa* that are deficient in QS occur naturally and are well known to cause infections (Schaber et al., 2004). Furthermore, *Pa* ΔlasR mutants in CF patients are associated with the progression of lung disease (Hoffman et al., 2009). The prevalence of *Pa* mutants lacking LasR is comparable to that of mucoid isolates, which are known to be one CF-adapted phenotype of *Pa* (Li et al., 2005; Hoffman et al., 2009). A study of 166 isolates from 58 CF patients found that 31% of them harbored lasR mutants. Hoffman et al. (2009) suggested that, like mucoidy, the inactivation of *lasR* in *Pa* is associated with the progression of CF lung disease. The mutants lacking QS may contribute to the progression of lung disease by positively affecting the virulence of other microbes, as demonstrated for *Ed* by the current study and by other studies of *A. fumigatus* or *C. albicans* (De Sordi and Mühlschlegel, 2009; Sass et al., 2019).

Growth inhibition of fungi by *Pa* has been suggested to result from several factors, e.g., the toxic effect of *Pa* metabolites (Holcombe et al., 2010; Bandara et al., 2013) or Fe limitation (Ferreira et al., 2015). The results of the present study describe a substantial growth inhibition. The growth inhibition was detected by planktonic co-culture growth assays and was also demonstrated in disk-diffusion assays, which achieved results comparable to those achieved by standard broth microdilution assays (Matar et al., 2003). While the live *Pa* cells exhibited growth inhibition in the disk-diffusion assay, culture filtrates did not affect *Ed* growth in stationary cultivation even though they showed a killing effect in planktonic culture. The main difference between disk-diffusion assays and planktonic growth assays in co-culture is the stationary cultivation associated with the prohibition of thorough intermixing of both strains of interest. Thus, lack of intermixing results in decreased concentration of culture filtrates and its molecules nearby fungal cells, implicating the difference in effects of culture filtrates in both included assays. Although growth inhibition was detected after 24 h and 48 h, biofilm formation was increased after 24 h, indicating that *Ed* biofilms exert a resistance mechanism against the killing effect of *Pa*. These resistance mechanisms could be explained by an overall protective benefit of biofilms against various molecules, among them therapeutic agents or host immune molecules (Mah and O’Toole, 2001). These mechanisms would allow the ECM to protect *Ed* cells within a biofilm from *Pa* toxins.

Previous *Ed* biofilm studies showed that maximal biofilm biomasses were achieved after 48 h (Kirchhoff et al., 2017). The higher biomass in *Ed* biofilms after 48 h than after 24 h was here inhibited when either *Pa* cells or culture filtrates were present. The time-dependent inhibition-effect indicates that the *Pa* QS molecules play a relevant role. Both AHL QS systems are known to be activated immediately after biofilm development has begun, reaching a peak at 48 h when a certain thickness of the biofilm has been achieved. Subsequently, QS gene expression decreases (De Kievit et al., 2001; Mangwani et al., 2015). It is assumed that after 48 h, the concentration of QS molecules is higher than that after 24 h due to the growth and biofilm formation of *Pa* over time. This may, explain the time-dependent biofilm behavior of *Ed* in the presence of *Pa.* The absence of both AHL QS systems reverses the inhibition effect, benefits the formation of *Ed* biofilms, and results in a higher biomass in biofilm cultures than in pure *Ed* biofilm cultures. Next to time-dependency, also *Pa* strain-dependency could be detected with increased *Ed* biofilm inhibitory effect by PA07 compared to PA14. It is known that different strains of *Pa* exhibit different phenotypical characteristics, including the regulation of QS (Chugani et al., 2012), explaining the strain-dependent effects.

It is known that the *in vitro* formation of *Pa* biofilms depends in part on the LasR QS system (Davies et al., 1998). The *Pa* biofilm culture filtrate exhibited an inhibitory effect comparable to that of the *Ed* biofilm culture filtrate, a finding leading to the assumption that the *Ed* QS molecules involved in the biofilm development processes are somehow working in a manner similar to that of the AHL QS molecules in *Pa.* Here, the AHL molecule 3-oxo-C12 HSL has been shown to inhibit *Ed* biofilm formation in a concentration dependent manner. While DMSO has been shown to drastically inhibit *Pa* biofilm (Guo et al., 2016), biofilm of *Ed* has not been affected this substantially. The Pa-biofilm reductive effect of 3-oxo-C12 HSL could not be detected as DMSO treatment resulted in already low values. However, to date no published studies have examined the QS molecules in black yeasts. Furthermore, for both species biofilm formation is higher under anaerobic conditions than under aerobic conditions. Unlike biofilm formation under aerobic conditions, fungal biofilm production under anaerobic conditions is enhanced in the presence of *Pa* after 48 h. The respiratory tract of CF patients exhibits hypoxic gradients, and it has been suggested that much of the CF airway is anaerobic (Costerton, 2002). *Pa* has been shown to grow equally well under either anaerobic or aerobic conditions (Worlitzsch et al., 2002), whereas the biofilms produced by *Pa* are more robust under anaerobic conditions than under aerobic conditions (Yoon et al., 2002). Generally, *Pa* expresses different genes under anaerobic conditions than under aerobic conditions.

The development of fungal biofilms is connected with filament formation and the impairment of hyphal differentiation is associated with inhibited biofilm formation processes (Blankenship and Mitchell, 2006). In *C. albicans*, the differentiation of hyphae has been found to be important during the intermediate phase of biofilm formation (Hawser and Douglas, 1994; Kojic and Darouiche, 2004). It has also been reported that certain *C. albicans* genes that are involved in biofilm formation are also necessary for hyphal development (Nobile and Mitchell, 2005). The morphology of dimorphic fungi is known to be influenced by various environmental factors, e.g., temperature and pH (Buffo et al., 1984).

We found that *Pa* exerts an effect on *Ed* morphology. The number and length of hyphae were decreased by *Pa* wild-type cells and filtrate. We detected an association between the pH of the media and morphology: filament formation increased in a more acidic environment when the fungus was grown under filament-inducing conditions. This finding supports those of Zhu et al. (2017) who showed that changes in pH influence morphologic transition in *Trichosporon cutaneum*. In contrast, for other fungi, e.g., *C. albicans*, acidic pH conditions result in a switch to yeast-like cells, whereas a weakly alkaline pH environment causes a switch to hyphal structures (Buffo et al., 1984). It has been shown that the consumption of carbon and nitrogen, among other molecules, may lead to the production of organic acid or other metabolites that cause an additional change in the pH of the media (Hugh and Leifson, 1953; Zhu et al., 2017). The Gram negative bacterium *Pa* produce acids via carbohydrate oxidation under aerobic conditions (Hugh and Leifson, 1953). Additionally, most bacteria produce alkaline substances in the presence of peptone (Hugh and Leifson, 1953). Both processes can influence the pH of the media. Because the carbohydrate oxidation is weak, the peptone metabolism often neutralizes the acidic effect (Hugh and Leifson, 1953). The impact of *Pa* metabolism on the pH of media has been shown to not be solely responsible for the inhibition of filament formation as the QS-deficient mutants did not affect *Ed* morphology as WT did.

*In vivo* survival of *G. mellonella* infected with *Ed* is negatively affected by culture filtrates of *Pa*; biofilm culture filtrates exert more of an effect than do planktonic culture filtrates. It is known that *Pa* is highly virulent in *Galleria*; a lethal dose of PA14 WT is 1 infected cell per larva, whereas a lethal dose of PA14 ΔlasR is 4 infected cells per larva (Jander et al., 2000). In this study, *Pa* exhibited strong killing effects with lethal doses of less than 10 cells per larva, resulting in death in less than 24 h. The sterile culture filtrates of planktonic and most biofilm *Pa* cultures did not exhibit a killing effect on larvae. In contrast, sterile and pure biofilm culture filtrates of the PA14 ΔrhlR mutant did exhibit killing effects on *Galleria*. Yoon et al. (2002) found a connection between accumulations of toxic nitric oxide as a result of anaerobic respiration and the lack of a working rhl QS system. The rhl QS system, together with nitric oxide reductase, is associated with the prevention of this accumulation. Nitric oxide is toxic to cells (Costerton, 2002); thus, the accumulation of nitric oxide may affect the survival properties of the insect larvae.

One limitation of our study may be that we used only a small number of strains. It may be of interest to study additional *Pa* strains with various phenotypes and sources to determine their effect on *Ed*. In this context, it may also be interesting to study the interactions between strains isolated from the lungs of a single CF patient, testing whether the microbes adapt to each other.

## Conclusion

Our study demonstrates that *Pa* exerts a large effect on *Ed.* The switch from the planktonic to the sessile lifestyle protects the black yeast-like fungus *Ed* from killing by *Pa*. The process of *Ed* biofilm formation is inhibited in the presence of *Pa*, partly by molecules of the AHL QS sytem in *Pa*. *Ed* filament formation is further inhibited by the presence of *Pa* because of the LasR and RhlR QS systems. The absence of the QS molecules results in enhanced formation of biofilm and filaments. In contrast, the virulence of *Ed* is significantly enhanced *in vivo* in the presence of *Pa* culture filtrates. In this study, the interactions between the two species are related to increased virulence potential of the fungus. The identification of molecules associated with these interactions as QS molecules of the AHL system is a possible target for novel strategies aimed at treating polymicrobial infections.

## Data Availability Statement

The datasets generated for this study are available on request to the corresponding author.

## Ethics Statement

All clinical samples used in the present study were analyzed after conventional microbiological diagnostic tests had been performed. The study did not include patient’s details and did not result in additional constraints for the patients. All data remained anonymous and were analyzed without patients’ consent because of the retrospective nature of the study. All analyses were carried out in accordance with approved guidelines.

## Author Contributions

LK and JS conceived and designed the experiments and wrote the main manuscript text. JS and P-MR supervised the work. A-KW, MS, AH, and US monitored larvae survival in the *in vivo* experiments. JB contributed the reagents, materials, and analysis tools. LK prepared all figures. All authors reviewed the manuscript.

## Conflict of Interest

The authors declare that the research was conducted in the absence of any commercial or financial relationships that could be construed as a potential conflict of interest.

## Abbreviations

AHL: N-acyl -L-homoserine lactone
ASM: Artificial sputum medium
CF: Cystic fibrosis
CFU: Colony-forming units
CLSM: Confocal laser scanning microscopy
C: concentration
CV: Crystal violet
DTT: Dithiothreitol
ECM: Extracellular matrix
Ed: *Exophiala dermatitidis*
MBE: Minimal biofilm eradication
OD: Optical density
Pa: *Pseudomonas aeruginosa*
PB: Phosphate-buffered
S: saline
QS: Quorum sensing
WT: Wild-type.
