# Supplementary material for: Phenotypical Characteristics of the Black Yeast Exophiala dermatitidis Are Affected by Pseudomonas aeruginosa in an Artificial Sputum Medium Mimicking Cystic Fibrosis–Like Conditions
Source: Front Microbiol. 2020 Mar 20;11:471. doi: 10.3389/fmicb.2020.00471 (PMC7100538; doi:10.3389/fmicb.2020.00471)
Supplement: Supplementary file 1 [file Data_Sheet_1.docx]

**Supplementary information**

**Phenotypical characteristics of the black yeast *Exophiala dermatitidis* are affected by *Pseudomonas aeruginosa* in an artificial sputum medium mimicking cystic fibrosis like conditions**

Kirchhoff L^1*^, Weisner A-K^1^, Schrepffer M^1^, Hain A^1^, Scharmann U^1^, Buer J^1^, Rath P-M^1^, Steinmann J^1,2^

*^1^Institute of Medical Microbiology, University Hospital Essen, University of Duisburg-Essen, Essen, Germany*

*^2^Institute of Clinical Hygiene, Medical Microbiology and Infectiology, Klinikum Nürnberg, Paracelsus Medical University, Nuremberg, Germany*

***Correspondence:**

Lisa Kirchhoff, Institute of Medical Microbiology, University Hospital Essen, University Duisburg-Essen, Essen, Germany

Telephone: + 49-201-723-3505

Fax: +49-201-723-5602

E-Mail: lisa.kirchhoff@uk-essen.de

Table S 1: pH values of 24 h incubated *E. dermatitidis* (Ed) cultures. Incubation at 35 °C and without agitation. Wt = wild type, S_ = sterile culture filtrate.

| **Sample** | **pH** |
| --- | --- |
| *Ed* pure | 7.2 |
| + PA14 WT | 8.0 |
| + PA14 ΔlasR | 8.0 |
| + PA14 ΔrhlR | 8.0 |
| + S_PA14 WT | 7.0 |
| + S_PA14 ΔlasR | 6.8 |
| + S_PA14 ΔrhlR | 6.8 |
| + 3-oxo C12 HSL (100 µM) | 7.4 |
| + DMSO (1 %) | 7.2 |


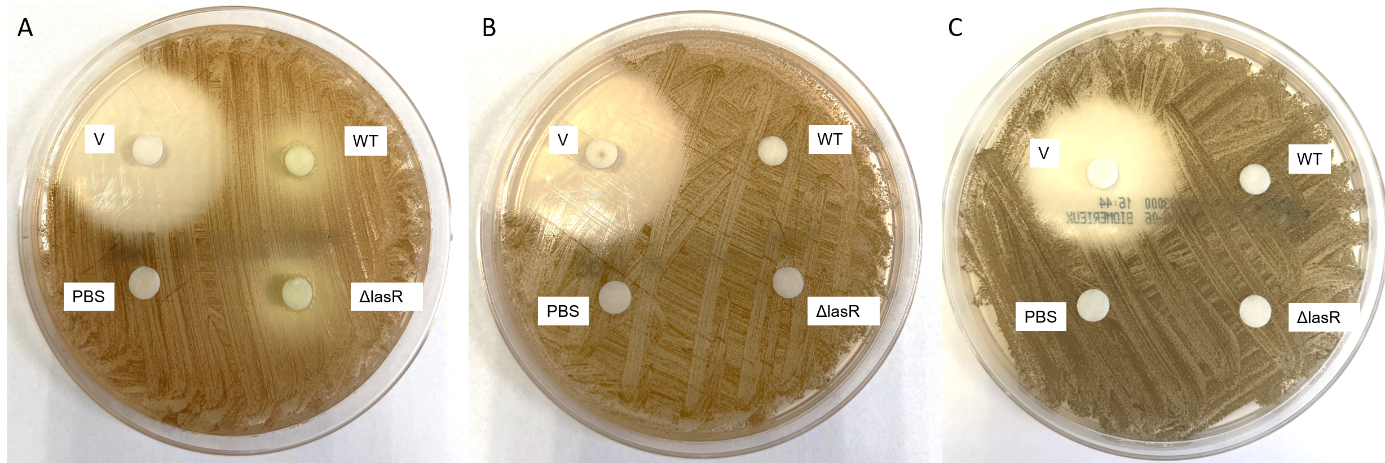
Fig. S1: Disk-diffusion assay of *E. dermatitidis* (P2) as the target strain and *P. aeruginosa* (PA14WT and PA14ΔlasR) as test strains. RPMI agar was used. Incubation for three days at 35°C. A: Viable *Pa*-culture. B: *Pa* planktonic culture filtrate. C: *Pa* biofilm culture filtrate. V = voriconazole, WT = PA14WT, ΔlasR = PA14ΔlasR).


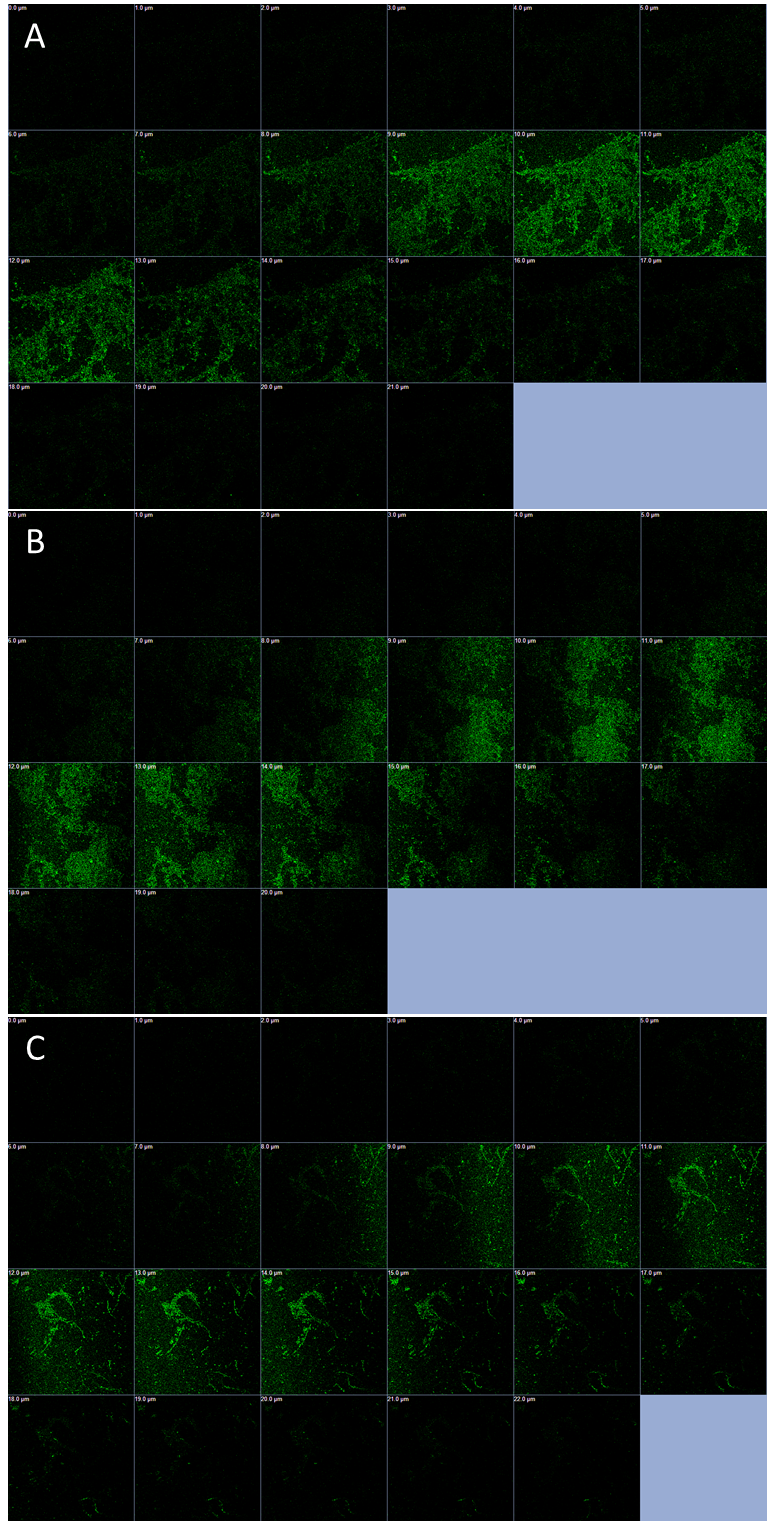


Fig. S2: Confocal laser scan microscopy images, Z-Stacks, of *E. dermatitidis* isolate P2 (CBS 116372) biofilm, formed in the presence (A & B) and absence of *P. aeruginosa* (PA07). Biofilm was grown for 24 (A) or 48 hours (B & C) at 35 °C in ASM. Biofilms extracellular matrix was stained by FilmTracer ™SYPRO® ruby biofilm matrix stain (Invitrogen) for 30 minutes, after stain was discarded sterile water was added for imaging. A laser with a wavelength of 405 nm was used. CLSM was done with a 40x objective.

Fig. S3: *Pseudomonas aeruginosa* wild-type (WT) strains PA07 and PA14, as well as two quorum-sensing (QS) mutants lacking LasR and RhlR biofilms formed after 24 h (grey) or 48 h (black) at 36°C in artificial sputum medium (ASM). Biofilms in mono- (pure) and co-culture with Exophiala dermatitidis (Ed). Biofilms were estimated by detachment of biofilm with 0.1% dithiothreitol and subsequent count of colony-forming units (CFU per mL).


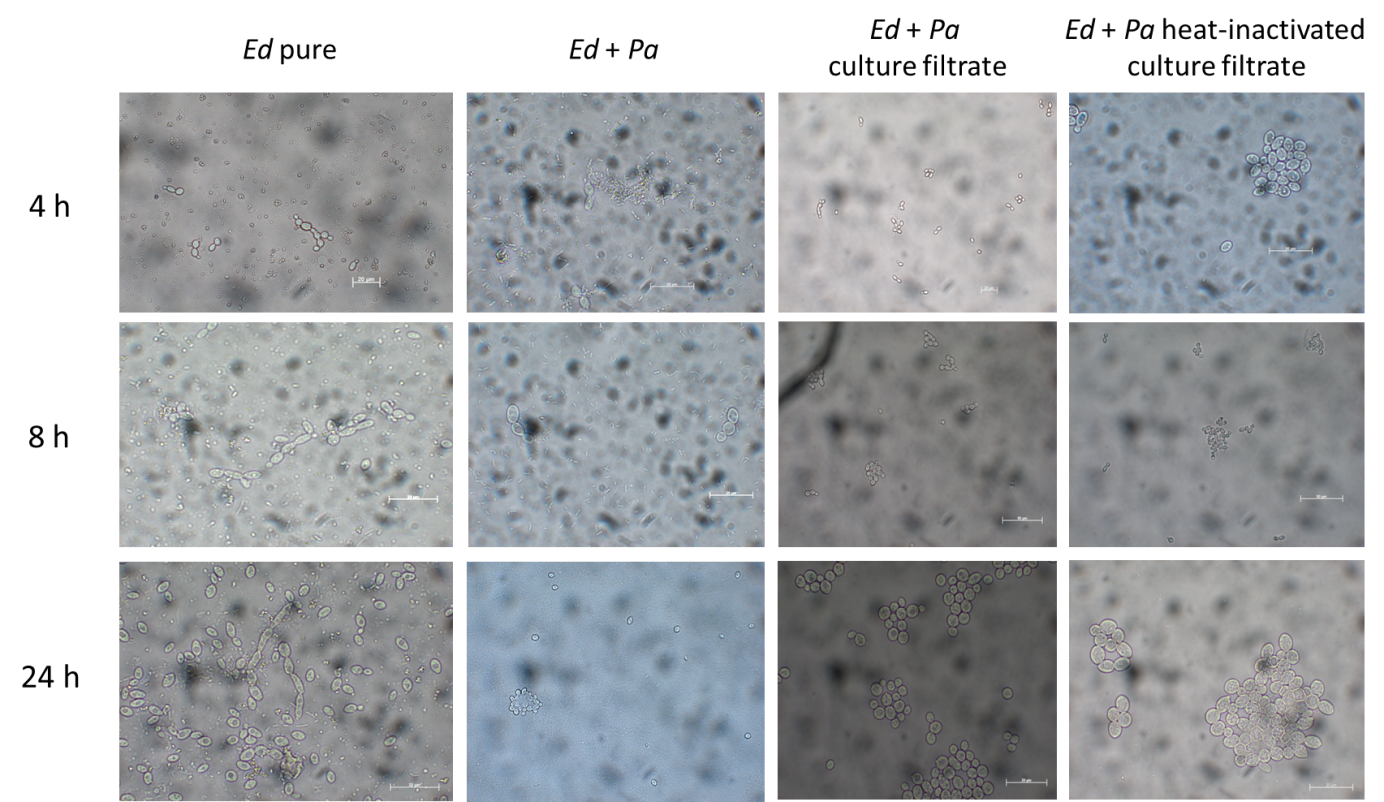


Fig. S4: *E. dermatitidis* (Ed, P2) in light microscope after 4, 8 and 24 h of incubation in ASM at 35°C. Grown in either pure culture (Ed pure), co-culture with *P. aeruginosa* (*Ed* + *Pa*; PA07) and in *P. aeruginosa* (heat inactivated) culture filtrate.

**
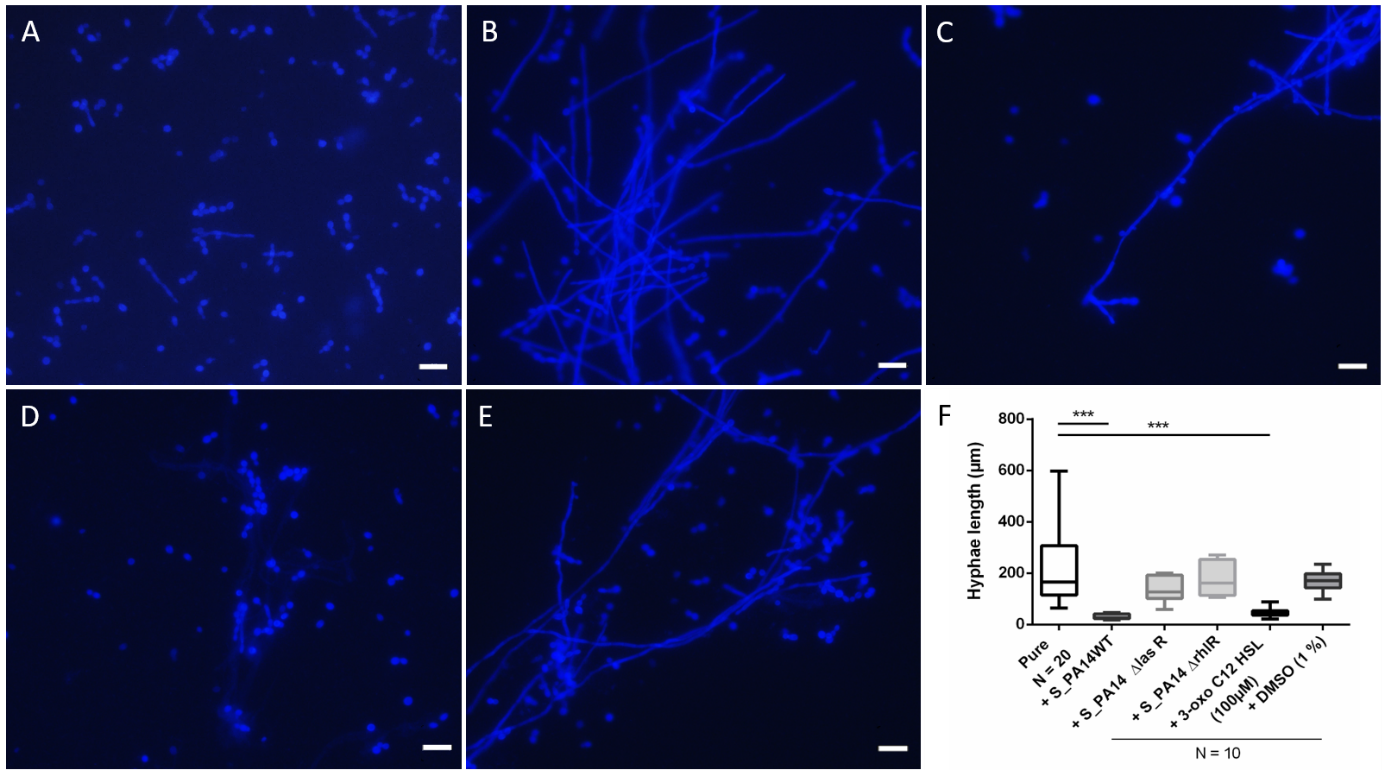
**

Fig. S5: *Exophiala dermatitidis* P2 morphology after 24 h of incubation in ASM with culture filtrate of *Pseudomonas aeruginosa* (PA14) WT (A), PA14 ΔlasR (B), PA14 ΔrhlR (C), with 3-oxo C12 HSL (100 µM, D) and with DMSO (1 %, E). Cultures were stained with calcofluor white and observed with a fluorescence microscope at 365 nm. Rulers indicate a length of 20 µm. F: length (µm) of hyphae of *E. dermatitidis.* Unpaired t-test, ***P < 0.001.


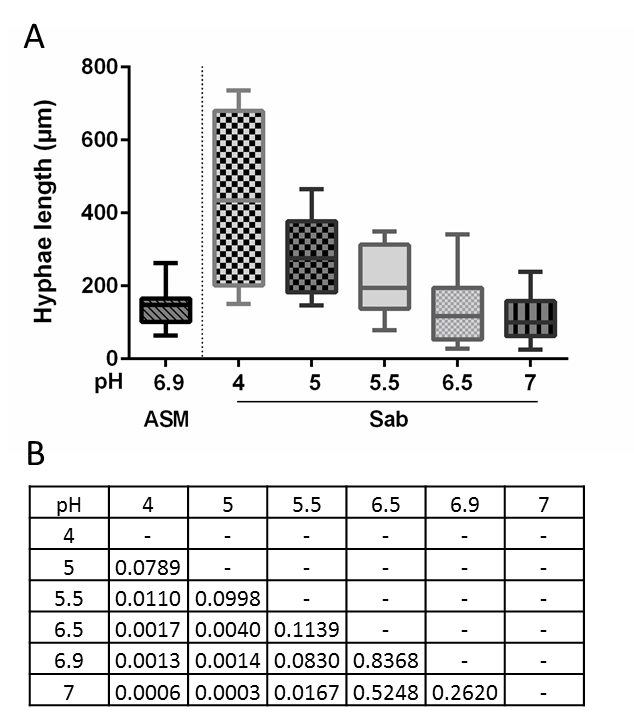


Fig. S6: A: *E. dermatitidis* P2 hyphae length after 72 h of incubation in Sabouraud (Sab) and artificial sputum medium (ASM) with varying pH. B: P-values estimated for significance testing with unpaired t-test. Cultures were stained with calcofluor white (BD) and observed in a fluorescence microscope (Zeiss Axio LabA1) at 365 nm. Images were taken with Axiocam.305 color and processed using Zen2coreV2.5 (Zeiss, Jena, Germany).


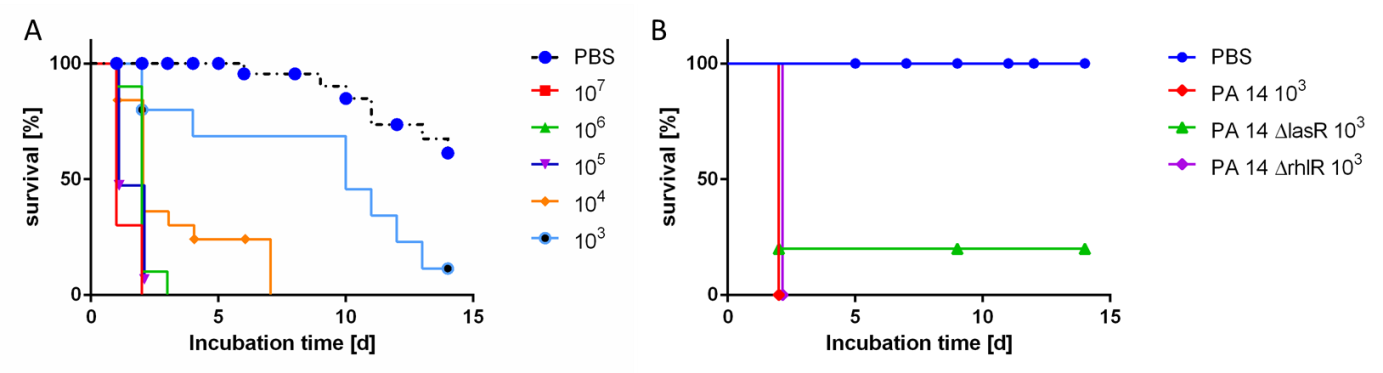


Fig. S7: Survival of *Galleria mellonella* after infection with *P. aeruginosa* strains PA07 (A) and PA14 (B) wild type (WT) and quorum sensing deficient mutants (ΔlasR and ΔrhlR), injected cell concentrations are varying from 10^7^ to 10^3^ cells/mL. As a control sterile PBS was used N = 30. Censored data are represented by symbols.





Fig. S8: Survival of *Galleria mellonella* after infection with *E. dermatitidis* (Ed; Strain P1) with and without *Pa* culture filtrate (S_PA) of strain PA07 (A/B), PA14 wt (C/D), PA14 ΔlasR (E/F) and PA14 ΔrhlR. As a control sterile PBS was used. A, C, E, G: planktonic culture filtrate was added to injection suspension (S). B, D, F, H: Biofilm culture filtrate (BFS) 24 and 48 h old, was added to injection suspension. N = 30. Log-rank (Mantel-Cox) test on significance: *: p < 0.05; **: p < 0.01; ***: p < 0.001; ****: p < 0.0001.





Fig. S9: Survival of *Galleria mellonella* after infection with *E. dermatitidis* (Ed; Strain CF2) with and without *Pa* culture filtrate (S_PA) of strain PA07 (A/B), PA14 wt (C/D), PA14 ΔlasR (E/F) and PA14 ΔrhlR. As a control sterile PBS was used. A, C, E, G: planktonic culture filtrate was added to injection suspension (S). B, D, F, H: Biofilm culture filtrate (BFS) 24 and 48 h old, was added to injection suspension. N = 30. Log-rank (Mantel-Cox) test on significance: *: p < 0.05; **: p < 0.01; ***: p < 0.001; ****: p < 0.0001.
